# The Impact and Cost of Scaling up GeneXpert MTB/RIF in South Africa

**DOI:** 10.1371/journal.pone.0036966

**Published:** 2012-05-31

**Authors:** Gesine Meyer-Rath, Kathryn Schnippel, Lawrence Long, William MacLeod, Ian Sanne, Wendy Stevens, Sagie Pillay, Yogan Pillay, Sydney Rosen

**Affiliations:** 1 Health Economics and Epidemiology Research Office (HE2RO), Department of Medicine, Faculty of Health Sciences, University of the Witwatersrand, Johannesburg, South Africa; 2 Center for Global Health and Development, Boston University, Boston, Massachusetts, United States of America; 3 National Health Laboratory Service, Johannesburg, South Africa; 4 Faculty of Health Sciences, University of the Witwatersrand, Johannesburg, South Africa; 5 Department of Molecular Medicine and Haematology, University of the Witwatersrand, Johannesburg, South Africa; 6 National Department of Health, Pretoria, South Africa; University of Cape Town, South Africa

## Abstract

**Objective:**

We estimated the incremental cost and impact on diagnosis and treatment uptake of national rollout of Xpert MTB/RIF technology (Xpert) for the diagnosis of pulmonary TB above the cost of current guidelines for the years 2011 to 2016 in South Africa.

**Methods:**

We parameterised a population-level decision model with data from national-level TB databases (n = 199,511) and implementation studies. The model follows cohorts of TB suspects from diagnosis to treatment under current diagnostic guidelines or an algorithm that includes Xpert. Assumptions include the number of TB suspects, symptom prevalence of 5.5%, annual suspect growth rate of 10%, and 2010 public-sector salaries and drug and service delivery costs. Xpert test costs are based on data from an in-country pilot evaluation and assumptions about when global volumes allowing cartridge discounts will be reached.

**Results:**

At full scale, Xpert will increase the number of TB cases diagnosed per year by 30%–37% and the number of MDR-TB cases diagnosed by 69%–71%. It will diagnose 81% of patients after the first visit, compared to 46% currently. The cost of TB diagnosis per suspect will increase by 55% to USD 60–61 and the cost of diagnosis and treatment per TB case treated by 8% to USD 797–873. The incremental capital cost of the Xpert scale-up will be USD 22 million and the incremental recurrent cost USD 287–316 million over six years.

**Conclusion:**

Xpert will increase both the number of TB cases diagnosed and treated and the cost of TB diagnosis. These results do not include savings due to reduced transmission of TB as a result of earlier diagnosis and treatment initiation.

## Introduction

South Africa bears a large share of the global burden of HIV and tuberculosis (TB) co-infection, with a TB prevalence of 795/100,000 in 2010 [1]. Recent studies have shown that up to 70% of TB suspects tested for HIV are HIV co-infected, with TB being the most common cause of mortality in HIV infected persons [2]. South Africa also has a high burden of multi-drug resistant TB (MDR-TB), with more than 7,000 cases diagnosed in 2010 [1].

In this context, conventional TB diagnostic technologies that have been used for decades, such as smear microscopy, are no longer reliable, because 24% to 61% of HIV-positive tuberculosis patients are smear-negative [3]. Significant hope for turning the tide of the TB epidemic thus lies with the recent development of rapid molecular assays. One of these, the GeneXpert System (Cepheid, Sunnyvale, CA) using the cartridge-based Xpert MTB/RIF (Xpert) assay, allows for rapid detection of *Mycobacteria tuberculosis* (MTB) and a rapid screen for rifampicin (RIF) resistance [4], [5]. Once a sputum sample has been collected from a patient, results are available in about 2 hours, without the requirement of highly trained laboratory personnel or additional biosafety measures [4].

In a multi-centre, prospective evaluation that included two South African sites, Xpert was found to be highly specific (99.2%) and highly sensitive (98.2% in smear-positive patients and 72.5% in smear-negative, culture-positive patients) for MTB [4]. Similar results have been obtained in other studies [2], [5]. In December 2010 the World Health Organization strongly recommended the use of Xpert, endorsing it as “the initial diagnostic test in individuals suspected of MDR-TB or HIV/TB”- in other words, most TB suspects in South Africa [6].

In March 2011, the South African National Department of Health announced a rapid, nationwide scale up of access to Xpert, to be achieved within a 2–3 year period. In conjunction with the South African National Health Laboratory Service (NHLS), it launched a pilot program that placed Xpert platforms in 25 smear microscopy laboratories across the country, with throughputs ranging from 16 to more than 400 tests per day and all instruments interfacing electronically with the centralised laboratory information system. By July 2011, over 50,000 samples had been processed, with a 3.4% error rate [7]. Based on this successful pilot, existing Xpert-enabled laboratories are now being upgraded to allow complete migration from smear microscopy to Xpert for TB diagnosis. Next, Xpert will be placed in all laboratories in nine designated high case-load districts. Finally, Xpert instruments will be placed at all other existing smear microscopy laboratories, fully replacing smear microscopy for the diagnosis of pulmonary TB in South Africa. Smear microscopy capacity for monitoring of TB treatment however will remain in these laboratories.

To help determine the additional budgetary resources required to procure and utilise Xpert as planned, while taking into account savings from the reduction in smear microscopy, the NHLS asked us to estimate the impact of Xpert scale-up on the number of TB cases diagnosed and treated and the incremental cost of the scale-up plan. Here we report the main results of our analysis, which have since been used to guide South Africa’s national policy.

## Methods

### Sources of Data

For this study we developed a model representing the diagnostic process starting with TB suspects, continuing to TB cases, and ending with treatment. The number of TB cases and cost were calculated quarterly for the financial years 2011 to 2016, covering the period from April 2011 to March 2017. Data for the model came from a random sample of all patients entered into the national-level NHLS TB specimen database in 2010 (n = 1,329,664) and a random sample of all patients entered into the national-level Electronic TB Register in 2010 (ETR, n = 286,741). Access to the two databases had been granted by the management of the NHLS and of the TB unit in the South African Department of Health, respectively. The databases had been anonymised for our use and did not contain any patient identifiers. We also used results of South African Xpert implementation studies [4], [5] and other literature [8]–[11]. Additional inputs on the performance of the Xpert test (RIF resistance rate and test failure rate) were based on NHLS data from the Xpert pilot phase during March and April 2011. Information on loss rates in suspects and baseline bacteriological coverage came from the Quarterly TB Statistics 2010 collected by the NDoH [12].

### Data from the NHLS Database

The national-level database of the National Health Laboratory Service provided model assumptions on the proportion of suspects who were smear microscopy-positive and those who were culture-positive (by smear status). The NHLS database contains the results of all laboratory tests done in the public sector in South Africa, with multiple entries for the same patient linked using the patient’s name, first line of address, and date of birth. We requested a subset of the database containing information on tests for TB (smear microscopy, TB culture, line-probe assay, and drug-susceptibility testing) of all patients who had an entry during the 2010 calendar year, as well as all TB test data on the same patients for 2004 to 2009. Due to the size of the original database we used a random sample of 100,000 patients (7.5% of all patients) for our analysis, representing all provinces and all types of clinical settings. Since the analysis was restricted to the diagnosis of TB in ambulatory care settings (and the South African guidelines prescribe a different approach to diagnosing patients in inpatient settings), we limited our analysis to the 83,977 subjects who contributed samples from outpatient clinics.

For positivity rates we used the first smear and/or culture performed in 2010 only. Since the database contained no information on whether tests were done for diagnostic or for treatment monitoring purposes, we deleted from the database everyone with a first entry in 2009 in order not to capture the treatment monitoring smears and cultures done during 2010 for patients initiated on treatment in 2009.

### Data from the Electronic TB Register

The Electronic TB Register (ETR) provided model assumptions on the proportion of patients with positive, negative, and unknown HIV status; the proportion of patients with a TB history; and proportions of smear and culture positive tests stratified by HIV and TB history status. The ETR is a register of all patients diagnosed with TB who are notified and initiated on treatment at the clinic level. The register starts at the clinic level as a book with one entry per notified patient. Copies of patient-level demographic and diagnostic information and treatment outcomes are sent to the district TB office several times a month, where they are entered into an electronic database. We downloaded information on all patients registered during the 2010 calendar year from all 9 provinces except KwaZulu Natal, for which data had been captured in a different format. We restricted our analysis to patients who had not moved or been transferred into the programme while already on treatment in order to avoid double-counting patients, and to patients with pulmonary TB. The remaining sample size was n = 57,688.

The database contained information for all patients on whether diagnosed cases were new cases or re-treatment cases and for some patients on HIV status. We used the proportion of re-treatment cases out of all cases to inform our assumption of the proportion of patients with TB history. In order to calculate the proportion of patients with a positive, negative or unknown HIV status we removed all patients with missing HIV information from the analysis (n = 32,951). For the analysis of proportions of smear and culture positive tests stratified by HIV status we regarded patients with an unknown HIV status (5.87%) as HIV positive, as suggested in the South African TB diagnostic guidelines [13]. Information on pre-treatment microscopy status was missing in 21.38% of patients.

### Scenarios and Algorithms

**Figure 1:**
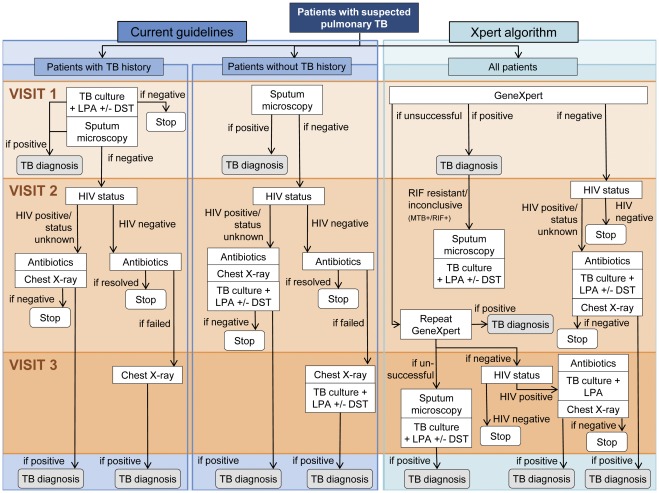
Diagnostic algorithm under current guidelines (Baseline scenario) and proposed new guidelines (Xpert scenario). LPA denotes line probe assay; DST, drug-susceptibility testing. Adult patients with suspected pulmonary MTB infection undergo a number of TB diagnostics at each of up to three consecutive diagnostic visits. Under the Baseline scenario, diagnostics are differentiated by whether or not patient have a history of TB treatment, and, for smear-negative patients, by HIV status. Under the Xpert algorithm, diagnostics are differentiated by HIV status for patients with a negative Xpert result only. Under both algorithms patients with a positive culture undergo further testing by line probe assay and, if this shows a resistance to RIF and/or INH, by drug-susceptibility testing for second line TB drugs. Under the Xpert scenario, patients with a positive Xpert result and RIF resistance (MTB+/RIF+) undergo further sputum microscopy and culture +/− LPA +/− DST for confirmation of the MDR-TB result and exclusion of XDR-TB. For smear microscopy, two sputa are collected; for an Xpert test, a single sputum is used. All tests are done on spot sputum samples.

**Figure 2:**
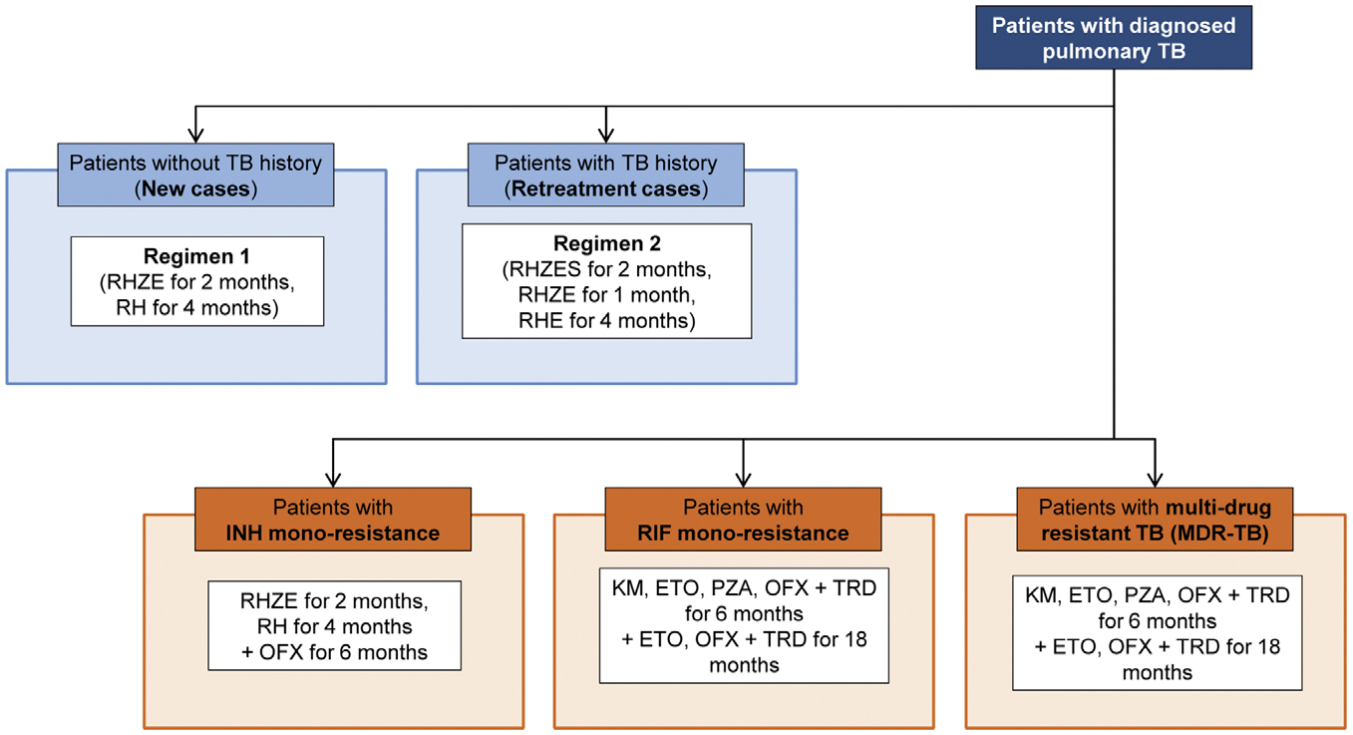
Treatment algorithm by resistance status. R denotes rifampicin, H, isoniazid, Z, pyrazinamide, E, ethambutol, S, streptomycin, OFX, ofloxacin, KM, kanamycin, ETO, ethionamide, PZA, pyrazinamide, TRD, terizidone. Patients diagnosed with pulmonary TB are initiated on TB treatment according to their drug resistance status and, for the Baseline scenario, by their TB history. In the Xpert scenario, all patients without resistance are treated as New cases; regimen 2 is no longer used.

The model follows quarterly cohorts of TB suspects through up to three diagnostic visits under either a baseline scenario (current guidelines) or an Xpert scenario, under which the Xpert technology is scaled up either by the end of 2012 (accelerated scale-up) or by the end of 2013 (gradual scale-up). Both scale-up scenarios start from an Xpert coverage of 16% in June 2011, representing the existing level of Xpert capacity after the pilot phase. Patients with a positive Xpert MTB diagnosis are assumed to be initiated on treatment according to their resistance status at the same visit as they receive the positive result. Under both scenarios, we count patients as diagnosed even if they fail to return for the visit at which the positive result would be communicated to them, though they would not be assumed to initiate treatment, and we assume no loss to follow up after treatment initiation since no data is available yet on whether and how loss to treatment would differ between diagnostic scenarios. The diagnostic algorithms used in the baseline and Xpert scenarios, which reflect South Africa’s current and proposed new guidelines for TB diagnosis, are illustrated in Figure 1 and the treatment options for diagnosed patients in Figure 2.

### Model Inputs and Assumptions

Table 1 lists the model assumptions and their sources and Table 2 the cost inputs.

**Table 1:** Model assumptions and sources.

Scenario	Value				Source
***Baseline scenario***					
*Proportions of TB suspects*	*All*	*TB history*	*No TB history*		
With TB history	0.20	−	−		ETR 2010
HIV+	0.56	0.54	0.57		ETR 2010
HIV −	0.38	0.41	0.37		ETR 2010
HIV unknown	0.06	0.04	0.06		ETR 2010
*Diagnostic status*	*All*	*TB history*	*No TB history*		
Proportion TB suspects sputum smear +^a^	0.06–0.10^b^	−	−		NHLS 2010
Smear +, HIV+	−	0.06–0.10	0.06–0.10		NHLS 2010^c^
Smear +, HIV−	−	0.05–0.08	0.06–0.10		NHLS 2010^c^
Proportion of TB suspects culture +	0.20	−	−		NHLS 2010
Culture +, smear +	1.00	−	−		NHLS 2010^c^
Culture +, smear −	0.08–0.13	−	−		NHLS 2010^c^
Culture +, smear −, HIV+	−	0.08–0.13	0.08–0.14		NHLS 2010^c^
Culture +, smear −, HIV−	−	0.06–0.11	0.07–0.12		NHLS 2010
Proportion of TB suspects diagnosed clinically^d^	0.01	−		−	Assumption
*Resistance*	*All*				
RIF mono-resistance rate	0.01				Matsoso 2010 [8]
INH mono-resistance rate	0.02				Matsoso 2010 [8]
RIF + INH resistance rate	0.09				Matsoso 2010 [8]
LPA sensitivity for RIF resistance	0.99				Parssons 2011 [9]
LPA sensitivity for INH resistance	0.88				Parssons 2011 [9]
*Visit inputs*	*All*				
Time between first and second visit	3–5 days				NDoH guidelines [13]
Time between second and third visit	6 weeks				Chihota 2010 [10]
Proportion cultures positive at second (third) visit	0.05 (0.95)				Chihota 2010 [10]
Proportion of clinically diagnosed TB suspects diagnosed at second (third) visit	0.5 (0.5)				Assumption
Loss to follow up after first visit	0.135				QTBS 2010 [12]
Loss to follow up after second visit	0.2582				Boehme et al 2011 (CT cohort) [5]
*Sample losses*	*All*				
Sample loss per sputum sample	0.01				NHLS 2010
Proportion of cultures contaminated	0.1				NHLS 2010
Proportion of contaminated cultures repeated	0.87				Assumption
***Xpert scenario***					
*Diagnostic status*	*All*	*Smear+*	*Smear-*		
Sensitivity for positive culture result, 1st test	0.80	1.00	0.79		M. Nicols (unpublished data); Boehme et al 2011 (CT cohort) [5]
Specificity for negative culture result, 1st test	0.99	−	−		
Failure rate, 1st test	0.03	−	−		NHLS Xpert data May 2011
Failure rate, 2nd test	0.03	−	−		Assumption
Proportion of TB suspects culture +					
of Xpert MTB+/RIF+ pts	0.99				M. Nicols (unpublished data)
of Xpert MTB+/RIF inconclusive pts	0.87				M. Nicols (unpublished data)
of Xpert unsuccessful twice, smear negative pts	0.06–0.11				Assumption (same as baseline rate)
of Xpert MTB-, HIV+ pts	0.05				Boehme et al 2011 (CT cohort) [5]
Proportion of TB suspects diagnosed by antibiotic trial and/or chest X-ray (Xpert MTB, HIV+)	0.01				Assumption (same as baseline rate for smear negative patients)
*Resistance*					
RIF resistance rate (mono-resistance and MDR)	0.07				NHLS Xpert data May 2011
Xpert RIF resistance sensitivity	0.90				Boehme et al 2011 (CT cohort) [5]
Xpert RIF inconclusive rate	0.02				NHLS Xpert data May 2011
Xpert RIF susceptible rate	0.91				Calculated from above
*Visit inputs*					
Loss to follow-up after first visit	0.133				M. Nicols (unpublished data)
Loss to follow-up after second visit	0.26				Boehme et al 2011 (all cohorts) [5]

ETR 2010, Electronic TB Register 2010; NHLS 2010, National Health Laboratory Services database 2010; NDoH guidelines, South African National TB Guidelines [13]; QTBS 2010, Quarterly TB Statistics, National Department of Health 2010 [12].

a Two sputa.

b The proportion smear positive amongst all patients, and the proportion culture positive amongst smear negative patients, decreases in both scenarios over time as a function of the growth in suspects, allowing us to model a stable epidemic.

c Adjusted using weights by HIV and history status from ETR.

d Smear negative, diagnosed by antibiotic trial and/or chest x-ray.

**Table 2:** Cost inputs in 2011 USD.

Cost item	Cost	Source
**TB diagnosis**		
***Baseline scenario***		
Sputum microscopy (fluorescent microscopy)	3	NHLS 2011 charges
TB culture (liquid medium, growth)	16	NHLS 2011 charges
TB culture (liquid medium, no growth)	12	NHLS 2011 charges
Line probe assay (LPA) for all positive cultures	24	NHLS 2011 charges
Drug susceptibility test (DST) (first-line drugs only)	72	NHLS 2011 charges, NHLS 2010 database
Chest x-ray	14	Public-sector charges
Antibiotic trial (amoxicillin and additional cotrimoxazole for PCP pneumoniafor all HIV+ patients)	2	Own data
Clinic visit: Nurse	9	Own data
Clinic visit: Physician	16	Own data
***Xpert scenario***		
*Instrument cost (desktop-computer model)*		
GX4	20,832	Cost analysis of NHLS pilot
GX8	54,077	
GX12	70,541	
GX16	86,919	
GX48	394,657	
*Recurrent cost per test*		
Total per test	32	
Cartridge (including shipping)	15–22^a^	
Staff	3	
Overheads	3	
Transport and logistics	2	
Calibration	1	
Training and quality assurance	0.5	
Consumables	0.4	
Waste disposal	0.3	
Sample collection	0.3	
**TB treatment per course (regardless of diagnostic scenario)**		
First-line treatment (non- resistant)	429	Drugs: Government drug depot information and South African TB Guidelines [13];All other: Sinanovic et al 2003 [11]
Second-line treatment (non-resistant)	823	
RIF monoresistance	3,280	
INH monoresistance	796	
Multi-drug resistance (outpatient care only)	3,280	
Inpatient care for MDR-TB (sensitivity analysis only)	20,530	

a In an agreement between the manufacturer and the Foundation for Novel Diagnostics (FIND), the cost of Xpert cartridges for the public sector in 116 high-burden and all low- and middle-income countries has been set to USD 16.86 apiece for volumes of between 600,000 to 1.7 million globally turned-over cartridges, USD 14.00 between for volumes between 1,700,001 to 3,700,000, and USD 10.72 for volumes from 3,700,001 cartridges onwards [14]. The cost used here includes the cost of shipping to South Africa and local value-added tax.

#### Number of suspects

Numbers of suspects (patients and/or contacts of TB cases with a positive TB symptom screen) were calculated using data on the general population aged 15 years and above from the Actuarial Society of South Africa AIDS Model [15] and an assumption of a prevalence of TB symptoms of 5.5% based on the Provincial Quarterly TB Reports [12]. In the main analysis, this percentage increases by 10% every year, in line with the targets for South Africa’s ongoing Intensified Case Finding campaign [16]. In sensitivity analysis we consider rates of 0% and 6.5%, a rate suggested by the WHO Stop-TB “Planning and Budgeting for TB Control” model for South Africa [17]. The smear positivity rate is set at 9.89% of suspects at baseline, based on the 2010 NHLS TB database, and, together with the culture positive rate of smear negatives, decreases in both the baseline and Xpert scenarios as a function of the growth in suspects, allowing us to model a stable epidemic. This rate of decrease is calculated as
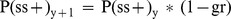
where P(ss+) is the proportion of patients who are smear-positive, y is the model year, and gr is the rate of growth in suspects (10%, 0%, or 6.5% according to growth scenario). (The same calculation applies to the proportion of smear-negative patients who are culture-positive).

The current bacteriological coverage rate (the proportion of suspects who have a documented smear microscopy result, based on the Provincial Quarterly TB Progress Reports [12]) of 85% is held constant in the baseline scenario but increases to 100% in the Xpert scenario to reflect a higher use of laboratory diagnosis as a result of the faster turn-around and greater specificity achieved by Xpert.

#### Cost data

We calculated the cost of TB diagnosis and treatment under the baseline and the Xpert scenarios from the government perspective, including the cost of outpatient visits, equipment, drugs, laboratory and radiological tests, infrastructure, training, and overhead. We used expert opinion and public-sector salary data to estimate the duration and cost of clinic visits, 2010 NHLS charges for all laboratory costs except the Xpert test, public-sector radiology costs, and public-sector drug costs and standard treatment algorithms for presumptive antibiotic treatment. TB treatment drug costs (including for mono-resistant and MDR-TB treatment) were calculated using August 2011 drug tender costs and the current South African TB guidelines [13]. All non-drug outpatient costs are based on previously reported estimates [11], adjusted for inflation to 2011 ZAR and converted to USD at 1 USD  = 7.94 ZAR [18]. We included only outpatient treatment costs for MDR-TB since during the projection period a new strategy that replaces inpatient with outpatient care for MDR-TB patients is planned to be rolled out [19]. Costs are reported in 2011 USD and presented undiscounted and inclusive of value-added tax (VAT).

The per-test cost of the Xpert technology was calculated in a separately-reported analysis [20], based on a bottom-up cost analysis of the pilot phase, estimates of the number and type of instruments required given diagnostic sample volumes at NHLS smear microscopy labs, and assumptions about when global volumes allowing discounts on the international price of Xpert MTB/RIF cartridges will be reached and how much of that global test volume will be borne by South Africa. Instrument costs were annualised over an expected useful life of the modules of four years; when varying the value to 3, 5 and 8 years, we found the cost per Xpert test to be insensitive to this assumption. The separately-reported analysis [20] estimated that in the period 2011 to 2013, the average cost per Xpert test performed will start at USD 33 and then decline to USD 25 between 2014 and 2016 when higher volumes and the resulting global volume discounts are reached. More detail on the cost per test can be found in Table 2.

#### Sensitivity analysis

Unless otherwise stated, all Xpert scenario results reported reflect accelerated scale-up, 10% growth in suspects, and South Africa procuring 50% of global Xpert test volumes [21]. In sensitivity analysis we considered variation in five parameters: a) 0% and 6.5% growth rate in suspects; b) South Africa’s share of global volumes at 90%; c) an impact of full Xpert coverage on smear positivity and culture positivity rates of suspects as a result of a reduction in transmission, based on the results of a previous model [22], d) South Africa accessing Xpert cartridges at the volume-discounted price of USD 10.72 ahead of global test volumes reaching 3.4 million in December 2011, and e) an additional 4 months of inpatient care per patient for MDR-TB, valued at the national average cost per inpatient-day equivalent of $168.28.

The study was approved by the Human Research Ethics Committee of the University of the Witwatersrand and the Institutional Review Board of Boston University Medical Center.

## Results

### Number of Suspects

Table 3 reports the projected numbers of suspects and patients diagnosed with TB under the baseline and accelerated Xpert scenarios. As a result of the 10% increase in suspects or contacts undergoing TB diagnosis expected from the ongoing Intensified Case Finding campaign [16], along with some population growth, the number of suspects requiring TB diagnosis increased in the model from 1.9 million per year to 3.2 million per year. Fifty-six percent of suspects were assumed to be HIV positive, based on data from the Electronic TB Register.

**Table 3:** Number of suspects and number of patients >15 years diagnosed with TB per year, by scenario.

		2011	2012	2013	2014	2015		2016		TOTAL			Avg. (% of total)
***All scenarios***													
All suspects		1,883,591	2,091,621	2,320,890	2,573,504	2,851,790		3,158,305		14,879,701			2,479,950 (100%)
- HIV+		1,062,910	1,180,302	1,309,678	1,452,228	1,609,265		1,782,231		8,396,615			1,399,436 (56%)
- HIV−		710,114	788,541	874,976	970,211	1,075,125		1,190,681		5,609,647			934,941 (38%)
- unknown HIV status^a^		110,567	122,778	136,236	151,065	167,400		185,392		873,438			145,573 (6%)
-smear positive		182,440	182,331	182,085	181,713	181,227		180,635		1,055,719			175,953 (7%)
-smear negative		1,701,150	1,909,291	2,138,805	2,391,791	2,670,563		2,977,670		13,823,982			2,303,997 (93%)
***Baseline scenario***													
Number of suspects diagnosed	335,930		339,433	342,709	345,814		348,801		351,724		2,064,411	344,068	
% of suspects	18%		16%	15%	13%		12%		11%		14%	14%	
Number of patients with drug susceptible TB	316,646		319,354	322,474	325,454		328,347		331,205		1,943,480	323,913	
Number of patients with drugresistant TB^b^	19,284		20,078	20,236	20,360		20,453		20,519		120,930	20,155	
% drug resistance^b^	4%		4%	4%	4%		4%		4%		4%	4%	
***Xpert scenario (accelerated scale-up, 10% growth in suspects)***													
Number of suspects diagnosed	369,054		412,133	446,985	457,852		469,357		481,633		2,637,013	439,502	
% of suspects	20%		20%	19%	18%		16%		15%		18%	18%	
Number of patients with drug susceptible TB	346,235		384,682	416,174	426,110		436,619		447,823		2,457,643	409,607	
Number of patients with drugresistant TB^b^	22,819		27,450	30,811	31,742		32,738		33,809		179,370	29,895	
% drug resistance^b^	6%		7%	7%	7%		7%		7%		7%	7%	
***Change between Xpert and baseline scenarios***													
Incremental suspects diagnosed	33,124		72,700	104,275	112,039		120,556		129,908		572,603	95,434	
% change	10%		21%	30%	32%		35%		37%		28%	28%	
% change in patients with drug susceptible TB	13%		28%	39%	41%		42%		44%		26%	26%	
% change in patients with drug resistant TB^b^	27%		51%	69%	69%		70%		71%		48%	48%	

a Suspects with unknown HIV status are assumed to be HIV positive, in keeping with the South African TB guidelines [15].

b “Drug resistant TB” includes INH mono-resistance, RIF mono-resistance, and multi-drug resistance.

### Number of Patients Diagnosed with TB

By 2013, the first year of full Xpert coverage, 30% more patients were diagnosed in the Xpert scenario than in the baseline scenario. This difference increased annually until 2016 due to the decreasing prevalence of smear-positive TB that resulted from intensified case-finding and the higher sensitivity of the Xpert algorithm for smear-negative TB. In addition, the Xpert algorithm led to a 69% increase in the number of patients diagnosed with drug resistance. The proportion of diagnosed patients with or without drug resistance who were initiated on treatment increased from 75% to 81%.

### Tests Used and Timing of Diagnosis

**Figure 3:**
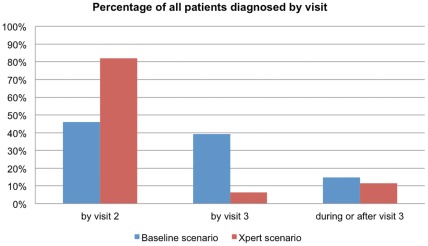
Timing of diagnosis for all patients. Percentage of all patients diagnosed by visit 2 (assumed 5 days after first visit), by visit 3 (assumed 4–6 weeks after first visit), and thereafter (accelerated scale-up, 10% growth in suspects).

**Figure 4:**
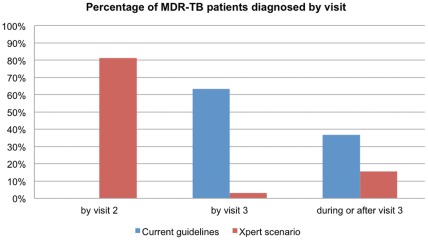
Timing of diagnosis for patients with drug resistant TB. Percentage of patients with drug-resistant TB diagnosed by visit 2 (assumed 5 days after first visit), by visit 3 (assumed 4–6 weeks after first visit), and thereafter (accelerated scale-up, 10% growth in suspects).

Under the baseline scenario 43% of patients were diagnosed by smear microscopy, 51% by culture, and 6% clinically. This breakdown changed dramatically under the Xpert scenario, in which 85% were diagnosed by Xpert, 12% by culture, 3% clinically, and only 0.05% by smear microscopy. Figures 3 and 4 show the distribution of diagnoses across visits. As can be seen, under the baseline scenario, 44% of all patients were diagnosed by their second clinic visit, three to five days after the first visit, and another 39% by visit 3, six to seven weeks after the second visit (Figure 3). At full Xpert coverage in 2013, 82% of patients were diagnosed by visit 2, three to five days after their initial presentation, and 91% by visit 3. The difference in timing of diagnosis was even more pronounced for MDR-TB patients, with 0% of patients diagnosed by their second visit under the baseline scenario and 82% under the Xpert scenario (Figure 4).

While Xpert can largely replace smear microscopy for TB diagnosis, it cannot yet be used as a substitute for microscopy in treatment monitoring, since a DNA-based molecular test such as Xpert is unable to distinguish between live and dead mycobacteria. As a result, all patients diagnosed at their first Xpert test as MTB+ have to undergo additional smear microscopy for the purpose of obtaining a baseline for treatment monitoring. The WHO recommendation for the use of Xpert, moreover, currently only applies to sputum samples, and not to smears done from other materials for the diagnosis of extrapulmonary TB. The need for some smear microscopy capacity thus remains, though the number of smear microscopy tests decreased by 61–67% at full Xpert coverage. Similarly, the number of diagnostic cultures declined by 21–23%, and the number of line-probe assays by 44–55%. The number of drug-susceptibility tests required, in contrast, increased by 52–65% at full Xpert coverage.

### Total Cost, Incremental Cost, and Cost Per Case

The incremental capital cost of introducing Xpert technology (including instruments, additional laboratory building space, security, and training) between 2011 and 2016 was estimated to be USD 22 million. The incremental recurrent cost (including cartridges, staff, transport, and quality assurance) over this time period varied between USD 287 million (gradual scale-up) and USD 316 million (accelerated scale-up). Capital cost did not differ between scale-up scenarios, but recurrent cost for the gradual scale-up scenario was lower in the first two years owing to the smaller number of machines placed and overall lower testing capacity. The resulting incremental cost for the Xpert roll-out per year was between USD 30 million and USD 64 million (accelerated scale-up) and between USD 20 million and USD 64 million per year (gradual scale-up).

Table 4 presents the resulting cost of the TB diagnostic programme (including laboratory cost, clinic visits, and clinical diagnosis) and of the diagnostic and outpatient treatment programme combined. At full Xpert coverage (from 2013 onwards) the Xpert technology increased the total cost of the TB diagnostic programme by between 53% and 57%, or USD 48 million to USD 70 million, per year. The cost per suspect tested increased by USD 21 to USD 22 per year, or between 53% and 57%. The cost per patient diagnosed with TB increased by between USD 46 and USD 52 per year, or 15% to 17%.

**Table 4:** Total cost of diagnostic and treatment programme and cost per case and per suspect by scenario [2011 USD].

	2011	2012	2013	2014	2015	2016
Diagnostic cost only (Accelerated scale-up, 10% growth in suspects, South Africa at 50% share of global volume)						
Annual cost						
Baseline scenario	74,094,947	81,908,809	90,497,431	99,950,392	110,355,101	121,807,536
Xpert scenario	115,149,393	131,577,329	138,644,077	154,645,352	172,283,949	191,721,257
Incremental annual cost	41,054,446	49,668,521	48,146,647	54,694,959	61,928,848	69,913,721
% change	55%	61%	53%	55%	56%	57%
Cost per suspect						
Baseline scenario	39	39	39	39	39	39
Xpert scenario	61	63	60	60	60	61
Incremental cost per suspect	22	24	21	21	22	22
% change	55%	61%	53%	55%	56%	57%
Cost per case diagnosed						
Baseline scenario	221	241	264	289	316	346
Xpert scenario	312	319	310	338	367	398
Incremental cost per case	91	78	46	49	51	52
% change	41%	32%	17%	17%	16%	15%
**Diagnostic and treatment cost** (Accelerated scale-up, 10% growth in suspects, South Africa at 50% share of global volume)						
Annual cost						
Baseline scenario	233,518,340	243,301,039	253,275,412	264,001,919	275,592,346	288,166,684
Xpert scenario	293,359,370	332,505,982	356,333,459	375,834,175	397,077,794	420,274,541
Incremental annual cost	59,841,031	89,204,943	103,058,047	111,832,256	121,485,448	132,107,857
% change	26%	37%	41%	42%	44%	46%
Cost per suspect						
Incremental annual cost due to MDR-TB	16,493,378	33,699,639	45,950,789	46,937,184	48,032,498	49,259,932
Baseline scenario	124	116	109	103	97	91
Xpert scenario	156	159	154	146	139	133
Incremental cost per suspect	32	43	44	43	43	42
% change	26%	37%	41%	42%	44%	46%
Cost per case diagnosed and treated						
Baseline scenario	695	717	739	763	790	819
Xpert scenario	795	807	797	821	846	873
Incremental cost per case	100	90	58	57	56	53
% change	14%	13%	8%	8%	7%	7%
**Sensitivity analysis**						
**Diagnostic and treatment cost**						
***Comparator:***Annual cost of Xpert scenario, accelerated scale-up, 10% growth in suspects, South Africa at 50% share of global volume, no impact on transmission under Xpert	293,359,370	332,505,982	356,333,459	375,834,175	397,077,794	420,274,541
Annual cost, 0% growth in suspects	292,161,355	319,454,961	329,074,987	331,720,629	334,173,849	336,446,725
% change	−0.4%	−3.9%	−7.7%	−11.7%	−15.8%	−20.0%
Annual cost, 6.5% growth in suspects	292,981,587	328,709,898	347,619,719	361,018,125	375,040,777	389,746,053
% change	−0.1%	−1.1%	−2.5%	−3.9%	−5.6%	−7.3%
Annual cost, SA’s share of global volume 90%	294,321,907	336,618,783	358,375,806	378,098,820	399,587,327	423,053,804
% change	0.3%	1.2%	0.6%	0.6%	0.6%	0.7%
Annual cost, assume impact on TB transmission and reduction of smear and culture positive rate under Xpert	293,359,370	332,505,982	343,222,009	347,974,800	358,497,984	373,431,446
% change	0%	0%	−3.7%	−7.4%	−9.7%	−11.2%
Annual cost, access to cartridge price @ 3.4 million tests by December 2011	283,221,432	282,951,630	329,113,173	375,834,175	397,077,794	420,274,541
% change	−3.5%	−14.9%	−7.6%	0%	0%	0%
Annual cost, including 4 months inpatient care for MDR-TB	564,792,450	709,986,080	803,618,350	826,971,995	851,905,168	878,696,873
% change	92.5%	113.5%	125.5%	120.0%	114.5%	109.1%

The outpatient cost of the TB treatment programme, assuming full coverage with treatment, increased by USD 55–62 million per year from 2013 onwards, or by 34–37%. This increase was the result of a rise in the number of patients initiating treatment due to lower loss to care during the diagnostic process. The cost of the full TB diagnostic and treatment programme per patient diagnosed and treated increased by USD 53–58 per year, or 7–8% (Table 4).

### Sensitivity analysis

In sensitivity analysis we found the annual diagnostic and treatment cost to be sensitive to the assumption of growth in the number of suspects (under full Xpert coverage, reduction in cost of 7.7% to 20% for 0% growth, and by 2.5% to 7.3% for 6.5% growth) and to assumptions about an impact of full coverage with Xpert technology on transmission via a reduction in the smear and culture positivity rates in suspects (reduction in cost of 3.7% to 11.2%) (Table 4). Results were not sensitive to changes in the assumption about South Africa’s share of the total Xpert test volume from 50% to 90%. Including an approximation of inpatient cost for MDR-TB, however, doubled the total cost of the Xpert scale-up due to the large number of additional cases of MDR-TB diagnosed by Xpert.

The accelerated scale-up scenario and an assumption of 50% volume share led to price reductions being realised earlier and to lower overall cost. Accessing the cartridge price that is conditional on 3.4 million accumulative cartridges sold ahead of time, i.e., in December 2011, saves 26% of total diagnostic and treatment cost over the first 3 years.

## Discussion

Based on the model we developed for the South African National Department of Health, we estimate that the introduction of Xpert technology will substantially increase the number of TB cases diagnosed, MDR-TB cases identified, and patients started on appropriate treatment. It will also accelerate diagnosis, with 82% instead of 44% of patients diagnosed after the first visit. While these results are clear improvements in the performance of the TB diagnostic programme, they come at a cost, with the annual cost of the diagnostic programme increasing by 53–57% annually at full Xpert coverage, or USD 48–70 million per year, and the annual cost of the treatment programme increasing by 34–37%. The projected total cost of TB diagnosis and treatment in 2011 under the accelerated Xpert scenario, USD 293 million, is 35% more than the USD 218 million estimated as the total public-sector TB budget for 2011 [1], although it comprises only 2% of the total public health budget of South Africa for that year [23]. Potential cost savings could come from the country’s guaranteeing to procure certain test volumes in order to access volume-dependent cartridge price discounts ahead of time, simultaneously ensuring access to Xpert technology at the lower price for all other countries.

The prospect of new molecular technology for the diagnosis of TB and MDR-TB with a potential for implementation at point of care gave rise to a number of papers urging that the operational difficulties of using this technology be taken into account alongside the standard cost and effectiveness considerations [22], [24]–[26]. Although it is impossible to foresee all the challenges that large-scale implementation of Xpert will generate, we attempted to incorporate these concerns into our model by capturing impacts and costs for up to three consecutive visits per patient and including patient and sample loss rates and test failure rates at every step. We also attempted to reduce bias introduced by trial-specific cost and selection by basing most epidemiological parameters on national-level operational data, rather than clinical trial results.

The analysis presented here is limited in several ways. First, our epidemiological model inputs are based on national-level databases which are designed for routine patient care and programme monitoring. Although this allows us to use data from a far larger patient sample than is available in clinical trials and observation studies, mismatching by the linking mechanism in the NHLS database or missing data in both databases could have introduced bias into our analysis. Since the resulting parameter values are similar to those reported by the national TB programme [1] we do not believe this bias to be large. Second, our analysis is restricted to the full and incremental cost of the new diagnostic algorithm only. As such, it does not fully capture the potential benefits of the Xpert technology, such as longer survival and better quality of life, or its opportunity cost. Third, since the baseline scenario reflects current guidelines for TB diagnosis, the frequency and cost of clinical diagnosis and of treatment initiation without laboratory diagnosis and the potential cost-saving if Xpert diagnosis prevents these patients from being treated might be underestimated. Fourth, we do not include loss to follow-up from treatment, which could be higher in the Xpert scenario as patients who would previously have been lost to follow-up during a longer diagnostic process might now be lost during the early stages of TB treatment. Fifth, as with any model of an intervention that has not yet been brought to scale, our results assume that the impacts and costs seen in the pilot phase of Xpert implementation in South Africa and in small demonstration projects will continue to reflect the impacts and costs when nationwide scale-up is achieved. While we have taken some potential variation into account in our sensitivity analyses, close monitoring of the scale-up will be needed to verify many of the assumptions we have made. Sixth, the algorithm for Xpert diagnosis might change in the years to come as a result of the experience generated during the roll-out. Total diagnostic costs may be reduced once greater confidence in the technology allows for a less conservative algorithm for the management of HIV-positive, Xpert negative patients to be implemented, or as a result of increasing test volumes if Xpert becomes the diagnostic of choice for paediatric TB suspects and suspects with extrapulmonary TB as well. Finally, the role of MDR-TB treatment in driving the total cost of the South African TB program is only partly explored in the model. Our results indicate that costs would nearly double if the existing inpatient model of MDR-TB treatment is not replaced with an outpatient model of care, a policy that has been approved but not yet widely implemented in South Africa.

Limitations notwithstanding, it is clear from our model that nationwide scale-up of Xpert MTB/RIF technology in South Africa will substantially increase the cost of the national TB diagnosis and treatment programmes. It will also vastly increase the number of TB cases diagnosed, MDR-TB cases identified, and patients initiating TB treatment. The importance of this potential benefit in helping to turn the tide against TB in South Africa has been recognised in policy, with the Minister of Health committing in May 2011 to roll Xpert MTB/RIF technology out “to every district in the next six months and to every facility in the next 18 months” [27]. As new data are made available over the course of the national scale-up, we will update the model presented here to continue to guide implementation decisions.
